# Dark-Channel Enhanced-Compensation Net: An end-to-end inner-reflection compensation method for immersive projection system

**DOI:** 10.1371/journal.pone.0274968

**Published:** 2022-11-09

**Authors:** Xiangmei Zhang, Zongyu Hu, Zhihong Wu, Hu Chen, Peng Cheng

**Affiliations:** 1 School of Computer Science, Sichuan University, Chengdu, China; 2 School of Aeronautics and Astronautics, Sichuan University, Chengdu, China; Karunya Institute of Technology and Sciences, INDIA

## Abstract

Immersive projection display system is widely adopted in virtual reality and various exhibition halls. How to maintain high display quality in an immersive projection environment with uneven illumination and the color deviation caused by the inter-reflection of light is still a challenging task. In this paper, we innovatively propose a deep learning-based radiation compensation for an L-shaped projector-camera system. This method employs complex reflection phenomena to simulate the light transport processing in an L-shaped environment, we also designed a Dark-Channel Enhanced-Compensation Net (DECNet) which composed of a convolutional neural network called Compensation Net, a DarkChannelNet and another subnet (such as sensing network) aiming at achieving high-quality reproduction of projected display images. The final output of DECNet is the compensation image to be projected. It is always a critical problem to establish appropriate evaluation and analysis indexes throughout the research of light pollution compensation algorithms. In this paper, PSNR, SSIM, and RMSE are proposed to quantitatively analyze the image quality. The experimental results show that this method has certain advantages in reducing the inter-reflection of the projection plane. And our method could also well replace the traditional process using the backlight transmission matrix. It can be concluded to a certain that this method can be extended to other more complex projection environments with strong scalability and inclusiveness.

## 1. Introduction

**Fig 1:**
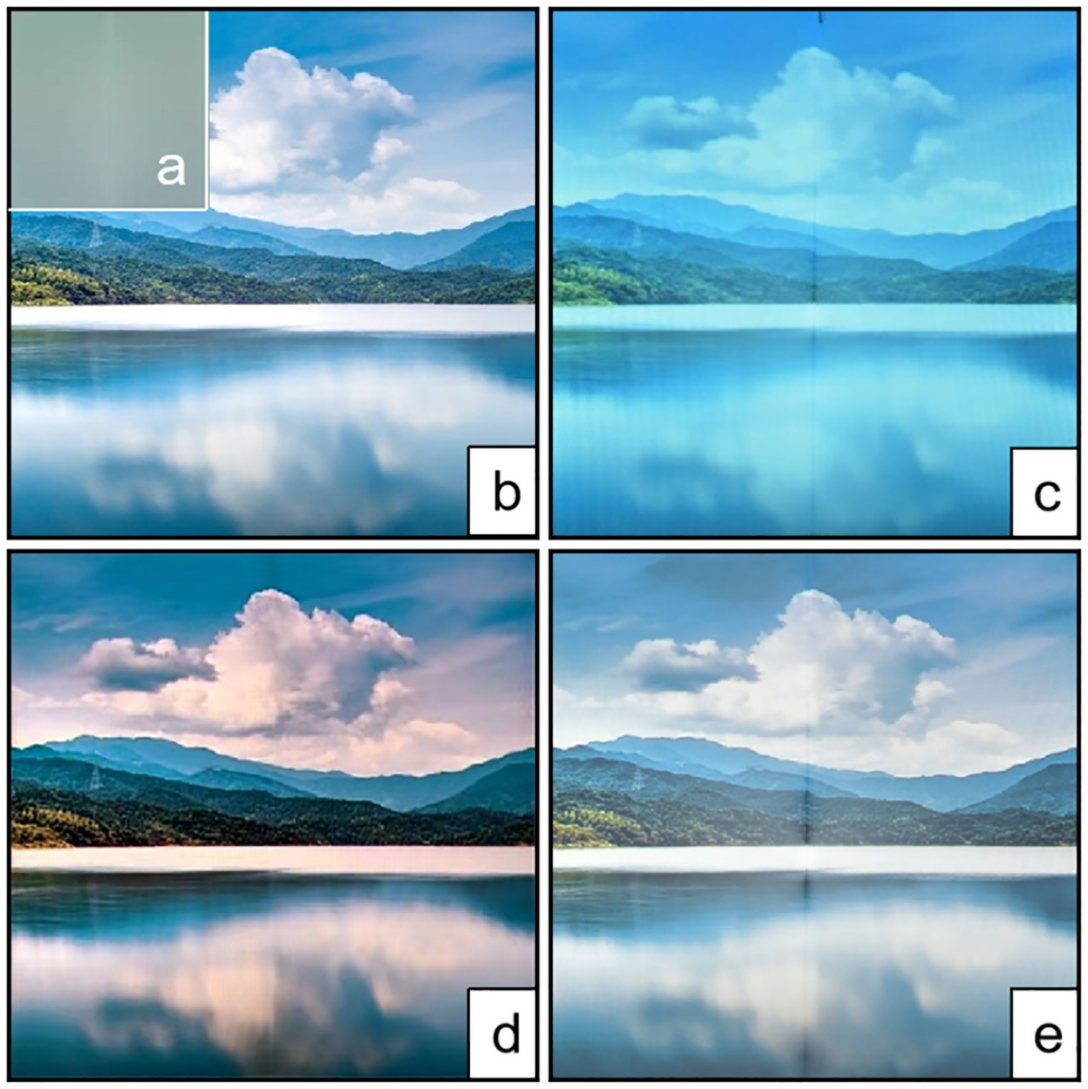
Radiometric compensation for L-shaped projecting screen based on Dark-Channel Enhanced-Compensation Net (DECNet). (a) White projection surface. (b) Original image. (c) camera-captured image when the original image is projected. (d) Compensated image. (e) camera-captured image when the compensated image is projected. (d) projected onto (a), compared (c) and (e), we are capable of finding (e) is closer to (b) with higher quality and less inter-reflection.

At present, projectors are tremendously widespread all over the world, especially in the large immersive and semi-immersive projection displays, which are widely adopted in Virtual-Reality (VR) and Augmented- Reality (AR) applications [1, 2]. In immersive VR system, the illumination uniformity and splicing correction are very important. It can directly affect the quality of the projected image. Almost all concerned problems, such as projection splicing fusion [3], geometric correction [3–7], and photometric correction (texture correction) [8–14] have been perfectly solved. However, problems such as luminance redundancy and the contrast degradation caused by complex optical phenomena have not been well addressed, as shown in Fig 1(c). In order to weaken the effect of inter-reflection, we propose an inter-reflection compensation method to compensate for the immersive projection screen and the associated complex photometric environment by modifying the input image of the projector. An example of inter-reflection compensation is illustrated in Fig 1.

**Fig 2:**
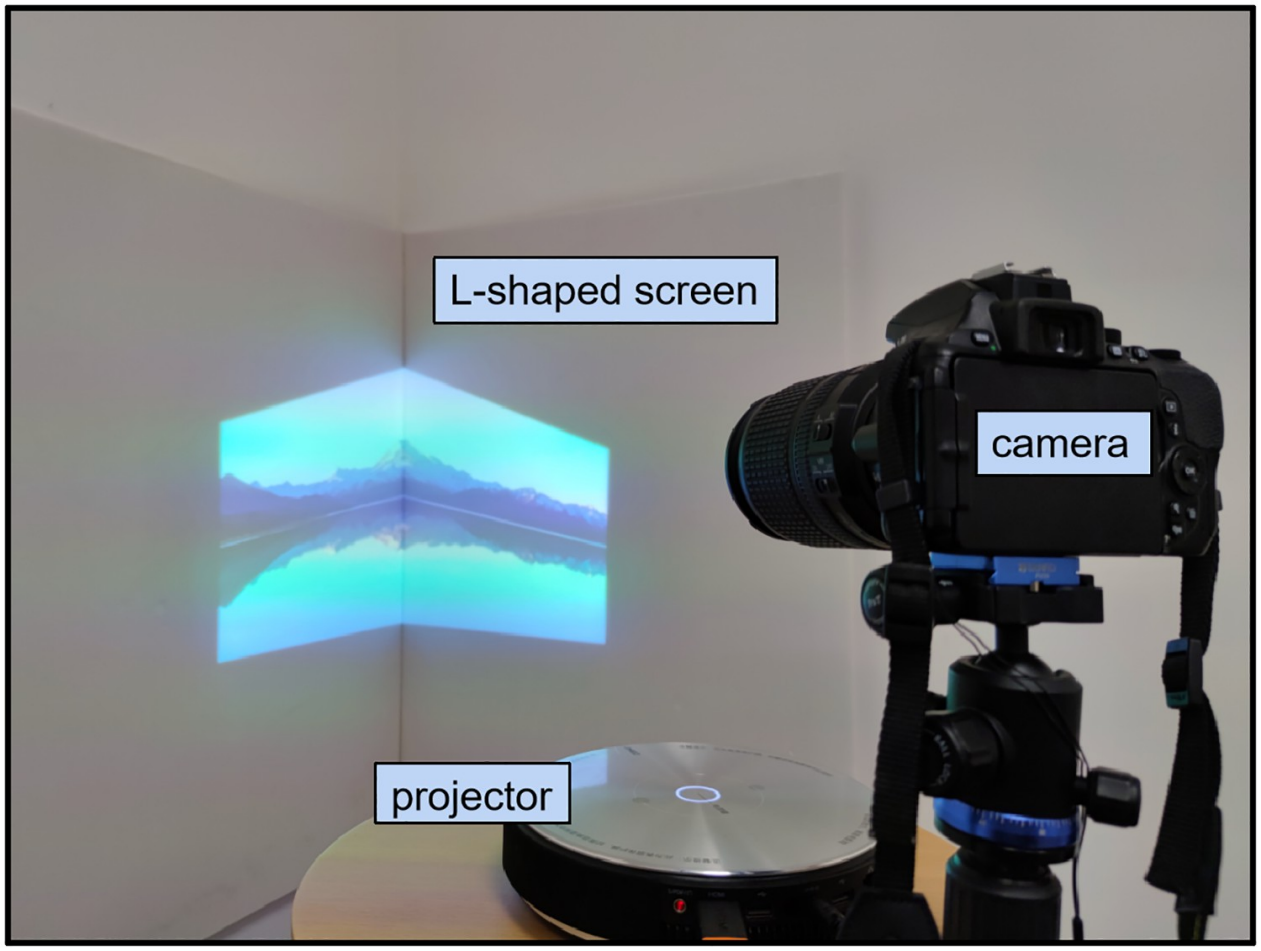
Projector and camera pair system. It consists of a camera, a projector, and an L-shaped projection screen which consists of two white screen.

To simulate the real immersive projection environment, we construct an immersive projection system which is consisted of a camera-projector pair and an L-shaped screen placed at a suitable distance and orientation, as shown in Fig 2. Firstly, the projector projects images to the L-shaped screen, and the environment lighting and inter-reflection will produce effects on the projection images according to the projection screen shape and material. Then, the radiometric transform, which is the process of mapping captured by the camera to input the image, should be determined. Previous studies [5, 11, 15–32] simulate this transforming process. However, due to the tremendous complexity of the photometric transmission, specular inter-reflection, and camera capturing process, it is extremely hard to accurately model the compensation explicitly.

In this paper, we proposed a convolutional neural network-based method named Dark-Channel Enhanced-Compensation Net (DECNet) for inter-reflection compensation in the immersive environment. We formulate the inter-reflection compensation problem to a deep learning task, which can learn the inverse process of implicitly light transmission matrix. The Dark-Channel Enhanced-Compensation Net consists of Compensation Net, Dark-Channel Net, and Perceptual-Loss-Net. The Compensation Net is able to extract the illumination information of the inter-reflection of the projection surface, and compensated images will be produced in the deep network layer in combination with the multi-level jump convolution layer. The DarkChannelNet can compensate for the brighter part of the inter-reflection of the image, can make the color of the image more saturated through the Dark-Channel algorithm. The Perceptual-Loss-Net can optimize perception loss and better reduce the difference between compensated images and original images. It is worth noting that DECNet is a direct image-to-image based compensation network and allows the compensation system to effectively and implicitly capture the complex inter-reflection during the projection process. Our survey found that there is very little research on the problem of inter-reflection directly through the image-to-image method.

To sum up, the method proposed in this paper has the following contributions.

1. In the immersive or semi-immersive projection system, DarkChannelNet is proposed to process the brightness information of the over exposed area of the image to make the projected image look undistorted.
2. The new compensation network DECNet is proposed, which uses three subnets to deal with different degrees of photometric information to compensate the problem of photometric nonuniformity caused by L-shaped screen.

In the second section, the related works on photometric compensation of the projection-camera system are introduced. In the third section, th model of illumination reflection of the projection-camera system and the network of radiometric compensation are established. In the fourth section, the training details are described. In the last two sections, relevant issues are discussed and draw conclusions.

## 2. Related works

Theoretically, the inter-reflection compensation process is an extremely complicated problem, which requires full consideration for the camera and the projector sensor radiometric responses, lens distortion, defocus, and the inter-reflection. Previous studies were mainly focused on designing explicit and precise mathematical inter-reflection models, which is extremely hard and computationally expensive. Generally speaking, this method can be roughly divided into the following two types: one is to establish the light transfer matrix in the scene [5, 11, 15–33], obtain the inverse matrix of the light transfer matrix through matrix inversion, and compensate for the original picture; the other is to establish the radiance model in the scene [10, 19, 34], the overall illumination radiance is calculated by inputting the intensity information of the light source and the reflection information of the scene to obtain the compensation picture.

### 2.1. Matrix based methods

Typically, inter-reflection compensation methods mostly depend on the light physical transport matrix, which can be measured by photoreceptor, laser beam, or other experimental equipment. It is generally assumed in these methods that a camera pixel is only influenced by its corresponding projector pixel, which means that there is an approximate one-to-one mapping between the camera and projector pair. Under this assumption, the nonlinear function relationship between the camera and the projector pixel can be described as a light transport matrix. Seitz et al. [31] proposed a method to inverse the light transport and provided an inter-reflection cancelation(IRC) operator to compute arbitrary bounces of light reflection. In their method, the transport matrix is acquired by a laser beam pixel by pixel. However, the scale of the light transport matrix is equivalent to the image resolution, which is typically huge and makes direct matrix inversion computationally intractable.

Ng et al. [21] proposed approximating the inverse of the light transport matrix systematically. This method utilized a smaller matrix to simulate the initial matrix and adopted the hierarchical inverse matrix to enhance the efficiency and accuracy of matrix inversion. Furthermore, the hierarchical inverse matrix were combined with Kajiya’s rendering equations to improve the computing efficiency [22]. A. Grundhofer and D. Iwai [35] proposed a thin-plate spline(TPS) based method, which proposes the thin-plates spline to approximate the matrix. Only 5^3^ = 125 sampling images are required for fitting the light transport function and further deal with clipping errors.

Apart from simplifying the acquisition and inversion process, the transmission matrix-based method is usually computational consuming and hard to perform in practice. Moreover, it is well known that a projection ray can illuminate several screen patches, and those patches can be illuminated by the inter-reflection of other patches as well. It means that only consideration of corresponding pixel pairs of the camera and the projector is not suitable for inter-reflection compensation.

### 2.2. Scene inverse irradiance method

To avoid tremendous computation and complexity experimental, a series of methods using scene inverse irradiance calculation have been proposed by researchers, such as utilizing the sparse characteristics of light transport matrix, separating the direct and indirect(global) components of reflections [19] and simulation-based solution [34].

O.Bimber [19] et al. proposed a compensation method of immersive projection system based on indirect scattering compensation calculation, which built an accurate mathematical model through known light source intensity, scene geometric information, screen reflection information and ambient light, The method calculated the inter-reflection directly, and the scatter matrix was proposed to compensate the images. However, the precise geometric information of the projection environment is necessary and hard to be obtained in some situations. YuLi et al. [34] proposed a physical multiplication-based method to compensate the inter-reflection in semi-immersive environment, which utilizes pro-cam system to simulate the physical multiplication. It is different from the iterative process of O.Bimber algorithm. The algorithm proposes an analytical method to solve the redundant inter-reflection in the scene. Therefore, it requires a large number of pictures, which is cumbersome and time-consuming. Whether iterative or analytical, the error may have a serious negative impact on the compensation quality in large scenes. Generally speaking, the algorithm based on optical transmission matrix usually needs to propose other auxiliary equipment to help realize the measurement of optical transmission matrix, which is difficult and costly in practice. This kind of algorithm usually needs to inverse a large matrix, and the accuracy of the algorithm is often not high, which is easy to lead to the low image quality of projection compensation.

To solve the problem mentioned above, we developed matrix free methods and proposed Dark-Channel Enhanced-Compensation Net (DECNet). DECNet is a direct image-to-image method without tremendous computation of reverse matrix and geometric calibration. It can fit the light transport matrix for compensating the images and improveing the visual quality of the projection images, especially the inter-reflection that causes image color distortion and high intensity for the whole immersive projection environment. Moreover, to adopt the human eye’s observation, we utilized Gamma-corrected [36, 37] on the train data, which can obviously weaken the inter-reflection in the immersive projection environment and acquire a better visual quality.

### 2.3. Learning based Dark-Channel method

The Dark-Channel a priori is an algorithm proposed by the Kaiming [38] in the field of defogging. His idea is that at least one of the three RGB channels of images based on no sky regions will have a low pixel value in a certain region. Based on this phenomenon, a definition of Dark-Channel is proposed. The Dark-Channel is used to obtain the transmittance and atmospheric illumination value, so as to realize the defogging function.

In this paper, in order to solve the problem of strong redundant reflected light caused by catadioptric projection, the general neural network is difficult to solve the problem of strong reflected light. Therefore, the learning-based Dark-Channel method is adopted to better solve the mutual reflected light caused by strong reflection in the image, and fuse with the network compensation image to make the result better.

## 3. Method

### 3.1. Problem formulation

**Fig 3:**
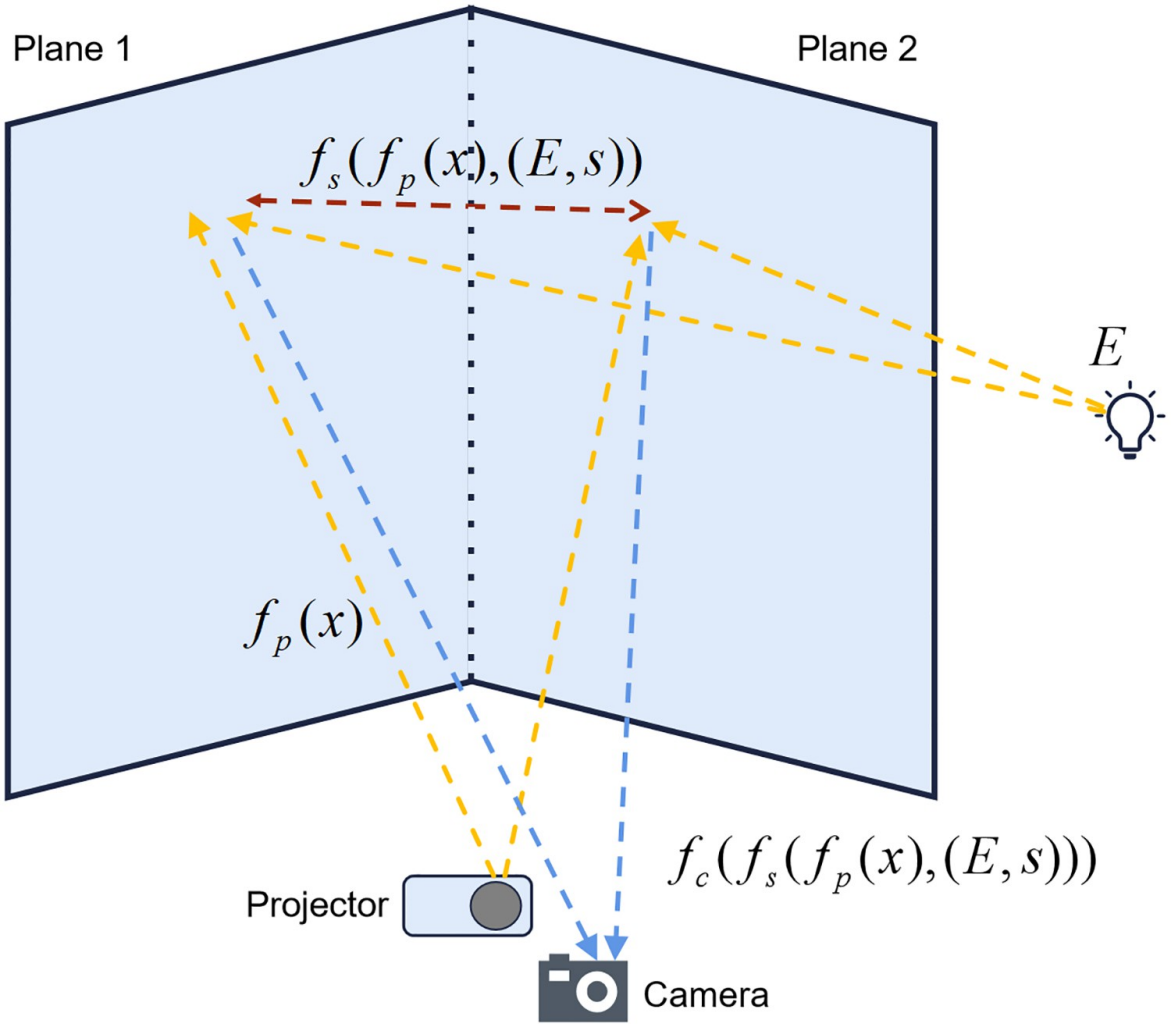
Light transmission process of L-shaped screen. The transmission process of light is affected by the radiation change of projector, and camera, surface reflection and overall illumination.

The purpose of inter-reflection compensation is to find a compensated image, which is able to reduce or even remove inter-reflection when it is projected. Our inter-reflection compensation system consists of an un-calibrated camera-projector pair and an L-shape projection screen placed at fixed distance and orientation, as is shown in Fig 3. The whole L-shape immersive projection system can be described as follows:

$$\begin{array}{c} I_{cam}=f_{c}\left(f_{s}\left(f_{p}\left(x\right),E,s\right)\right) \end{array}$$ (1)

Where *f*_*p*_ and *f*_*c*_ are the projector’s and the camera’s composite radiometric transform function respectively, *f*_*S*_ is the surface reflectance function, *s* is for different screen materials and denotes different surface spectral reflectance property. *E* is the influence of global lighting irradiance distribution. In our work, images are obtained in the dark environment, while *E* is ignored and set to 0. Our purpose is to find an input image x*, which is called compensation image for image *x*, and make the camera-captured image *y* similar to the input image *x*, that is *y* ≈ *x*. The above equation can be transformed to be:

$$\begin{array}{c} x=f_{c}\left(f_{s}\left(f_{p}\left(x^{*}\right),E,s\right)\right) \end{array}$$ (2)

To simplify the formulation, we define the whole radiation and spectral reflectance transform function in 2 as *T* and it can be expressed as:

$$\begin{array}{c} x=T\left(x^{*},s\right)\Rightarrow x^{*}\Rightarrow T^{-1}\left(x,s\right) \end{array}$$ (3)

Therefore, our goal is to find the inverse transform matrix *T*^−1^ to generate compensated image x*. In our work, we utilize a set of image pairs of $\chi ={\left\{\left(x_{i},y_{i}\right)\right\}}_{i=1}^{N}$ to learn the light transform matrix, where *x*_*i*_ and *y*_*i*_ represent the ground truth image and camera capture image respectively. Then, with the loss function and the learnable network parameters *θ*, the formulation of the learning process can be described as follows:

$$\begin{array}{c} y=T\left(x,s\right)\Rightarrow x\Rightarrow T^{-1}\left(x,s\right) \end{array}$$ (4)

$$\begin{array}{c} \theta =argmin\sum_{i}Loss\left(T_{\theta }^{-1}\left(y,s\right)-x_{i}\right) \end{array}$$ (5)

Traditionally, the transform matrix *T* and the inverse matrix *T*^−1^ should be figured out. However, the transform matrix of *T* the radiation and inter-reflection in an immersive projection environment is very complex and maybe invertible. It is hard to obtain the inverse matrix *T*^−1^. A direct image-to-image manner is proposed in our method to utilize a convolution neural network to avoid calculating *T*^−1^.

### 3.2. Network structure

**Fig 4:**
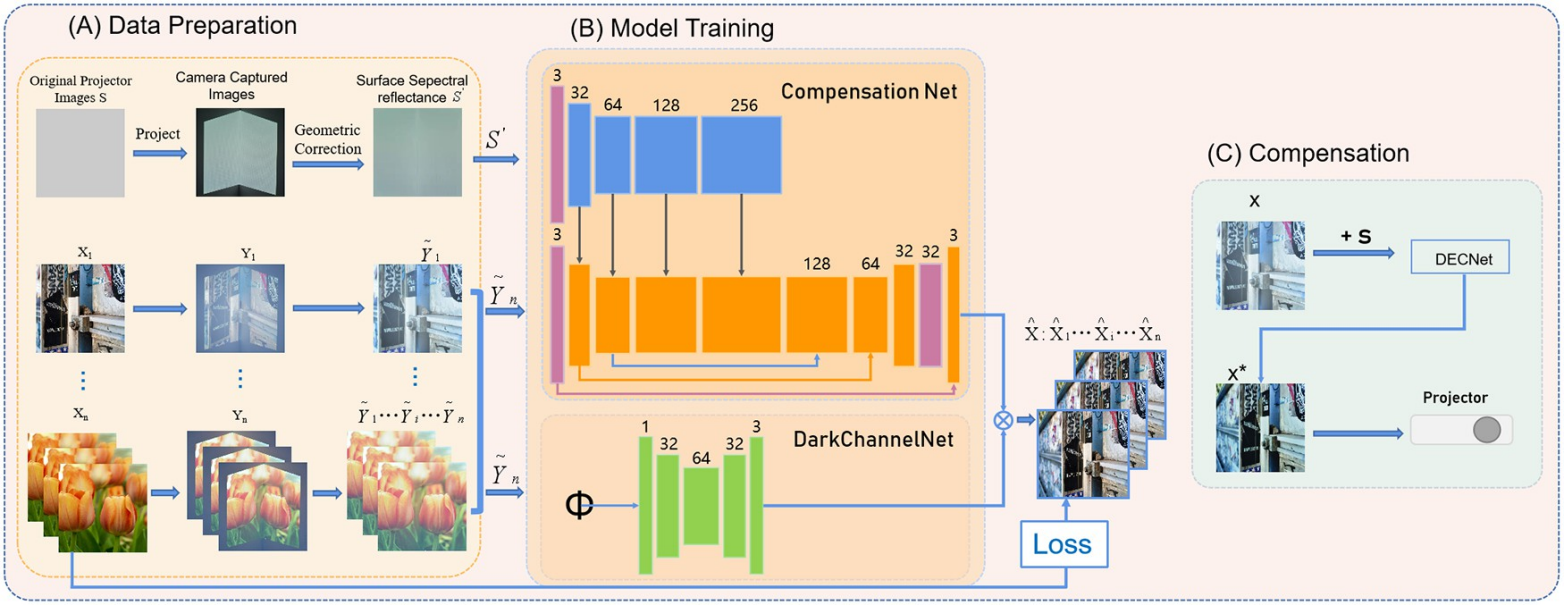
Light transmission process of L-shaped screen. The transmission process of light is affected by the radiation change of projector and camera, surface reflection, and overall illumination. (A) The project captures a surface image and a set of sampled images. (B) DECNet Model. (C) By training the model, the input image y can be compensated and projected. The architecture of the DECNet (the relu layer is omitted). All convolution layers consist of 3 × 3 filters and all transposed convolution layers are composed of 2 × 2 filters composition. Both the upper sampling layer and the lower sampling layer propose two steps. The number of filters in each layer in the figure is marked on its top. The skip convolution layer is displayed in the color arrow, and the number of layers and filters is marked as # layer # filters. *ϕ* representing the image calculation process of the dark channel.

As shown in Fig 4, the flowchart of the proposed projector compensation is consisted of three major steps. And the Network Structure’s details are described as follows.

1. Data preparation

The input is image pairs of 512 × 512 RGB, which are respectively camera-captured projection images and pure grayscale pictures that can provide some illumination. Since the human eye’s sensitivity for the intensity is not a linear response, if the original image is directly proposed for training, the training compensation effect will be reduced or high-quality inter-reflection compensation images cannot be obtained. In order to enhance the robustness of the model and the convenience of the algorithm, Gamma-corrected is proposed to solve this problem. Gamma-corrected is the method of editing the Gamma curve of the image and linear tone editing of the image to detect the deep part and shallow part of the image signal. Through Gamma-corrected [36, 37], the train images are much closer to those in the human eye’s sensitivity.

2. Network

The DECNet consists of Compensation Net, Perceptual loss network, and Dark Channel Net. Compensation Net is inspired by the work of CompenNet [10]. The network architecture is shown in Fig 4. Compensation Net is designed with two input images, the uncompensated image captured by the camera and the pure white image captured by the camera. The Compensation Net is composed of a backbone-network and branch-subnet similar to Unet. The branch network extracts features through a DetailModule (purple rectangular box) and a series of down-sampling, and then combines the sample layer with the pixel layer. In addition, the network is allowed to learn global illumination and inter-reflection, and finally add the extracted features to the corresponding backbone. In the backbone network, the skip convolution layer is proposed to transfer the low-level interaction information to the high-level feature map. Finally, we propose two transposed convolution layers to gradually sample the future mapping to obtain the output image. At the beginning and end, a DetailModule is added respectively to generate more detailed feature information. DetailModule (purple rectangular block), consisting of a 3 × 3, GroupNorm, ChannelAttention, and SpatialAttention. 3 × 3 convolution is to increase the local context information and effectively increase the receptive field. By adding grouping normalization and attention mechanism, DetailModule can better extract the edge detail information and image bottom detail features in the projected image, so as to enrich the color of the generated image.

To increase the visual quality of the projection image, we add the Perceptual-Loss-Net [34] to the Compensation Net architecture. The main principle of perceptual loss is to extract the feature information in the feature domain rather than in pixel domain. During the training process, perceptual losses measure image similarities are more robust than per-pixel losses, and at test-time the transformation networks run in real-time. In our paper, VGG16 network is proposed to extract both low-level and high-level features for perceptual loss. Moreover, to optimize the network in both domains, L1+L2+Lssim loss ensures the overall quality, and the perceptual loss enhances the sharpness of the image and the color saturation.

In order to further solve the problem of strong inter-reflection caused by L-shaped screen, we use a DarkChannelNet [38] to increase the saturation of the image and the uneven part of the image due to the depth of field. Make the best effect through a learnable network. The DarkChannelNet is composed of an Unet like structure with residual connections. It samples the input image directly, calculates the dark channel, takes the most value from the three RGB channels to form a gray-scale image, and then fills the dark channel with 1 and subtracts itself. The best effect is achieved through the learnable network, and the initial value of the weight is set to 0.85. We can see that the results after adding a learnable dark network can better reflect the color brightness and saturation of the image than those without this network.

### 3.3. Loss function

Generally, L1, L2, or L*ssim* loss functions are proposed for paired image-to-image translation work. However, these losses or their combination are not enough to restore the high-frequency information of the ground reality, which may lead to the blurring of the human eye experience in the immersive projection environment. In order to preserve the high-frequency information of the image, we propose a loss method combined with perceptual loss. Perceptual loss refers to the propose of high-level global information and low-level detail information for texture reconstruction. By selecting the feature output of a certain layer and inputting the perceptual loss function, the details can be enhanced and the high-frequency information of the image can be maintained. Here we propose vgg16 as the perceptual loss to reconstruct high-frequency details. Therefore, we combine L1, L2, L*ssim*, and L*perceptual* loss functions to train the model and preserve the high-frequency information and color saturation of the generated image as much as possible.

The final loss function of our method can be formulated as follows, where λ controls the effects of perceptual loss. Since the different λ leads to different effects on the quality of the generated images and there is a high demand on the color saturation in the immersive projection system, we take a low perceptual loss to avoid the color distortion and set it to be 0.01:

$$\begin{array}{c} LOSS=L1+L2+L_{ssim}+\lambda L_{perceptual} \end{array}$$ (6)

The formulation of perceptual loss can be described as follows:

$$\begin{array}{c} L_{perceptual}=\sum_{i=0}^{n}MSE\left(\varphi \left(y_{i}\right)-\varphi \left(T\left(x_{i}\right)\right)\right) \end{array}$$ (7)

where *x*_*i*_ is the camera-captured images, *y*_*i*_ is the ground truth pictures, *φ* refers to VGG16 feature extractor from 3th and 5th max-pooling layers and *T* denotes the Compensation Net.

## 4. Benchmark and experiment detail

### 4.1. System configuration and dataset

Our immersive projector-camera system consists of a Nikon DX VR camera with a resolution of 2992 × 2000, and a JMGO G7 projector with the projection resolution of 1920 × 1080. The distance between the camera and the projector is 100 mm, and there is an L-shape screen in front of the projector with a distance of 250mm. The camera’s white balance mode, shutter speed, ISO and the focus are respectively set to Auto, 1/90, 200, and f = 5.6. To simulate the real immersive projection system, we captured the pictures in the dark to exclude the influence of global lighting.

Since there is no corresponding public data set for immersive projection system research, we capture the images with a camera to construct cam-cap data set, including 3000 512 × 512 RGB images for training and 300 images for validity. For the projection images on the folding screen are always accompanied by the geometric distortion and can’t be used as the data set directly, we utilize a geometric correction in the camera-captured images. The ORB detector is used to find feature pairs between the ground truth image and the image captured by the camera on the left and right parts. Random Sample Consensus filters out wrong matches and calculates the homograph matrix to align the distorted images in both the left and right parts [5]. Then, Gamma-corrected was performed on those pictures, where the Gamma value is 2.2 [39]. To facilitate the image’s capturing, the images of the camera-captured and the ground truth images were down-sampled to the size of 512x512.

### 4.2 Implementation details

We trained our model under the PyTorch framework for 30000 iterations with a batch size of 2. Adam function was used as the optimization function with the learning rate increasing from l^*e*−3^ to l^*e*−4^ gradually. Furthermore, in order to find the best weight of perceptual loss, different λ values of 1, 0.1, 0.01, 0.001 were respectively evaluated. The influence of those different λ values will be illustrated in section V in detail.

## 5. Discussion

### 5.1. Comparison with state-of-arts

On the evaluation benchmark, we compare DECNet with four kinds of inattentive guidance methods, the traditional inter-reflection compensation method O.Bimber method, the CompenNet model and the improved CompenNet model CompenNet++. Meanwhile, we compared the effects of no Gamma and no Dark-Channel networks.

**Fig 5:**
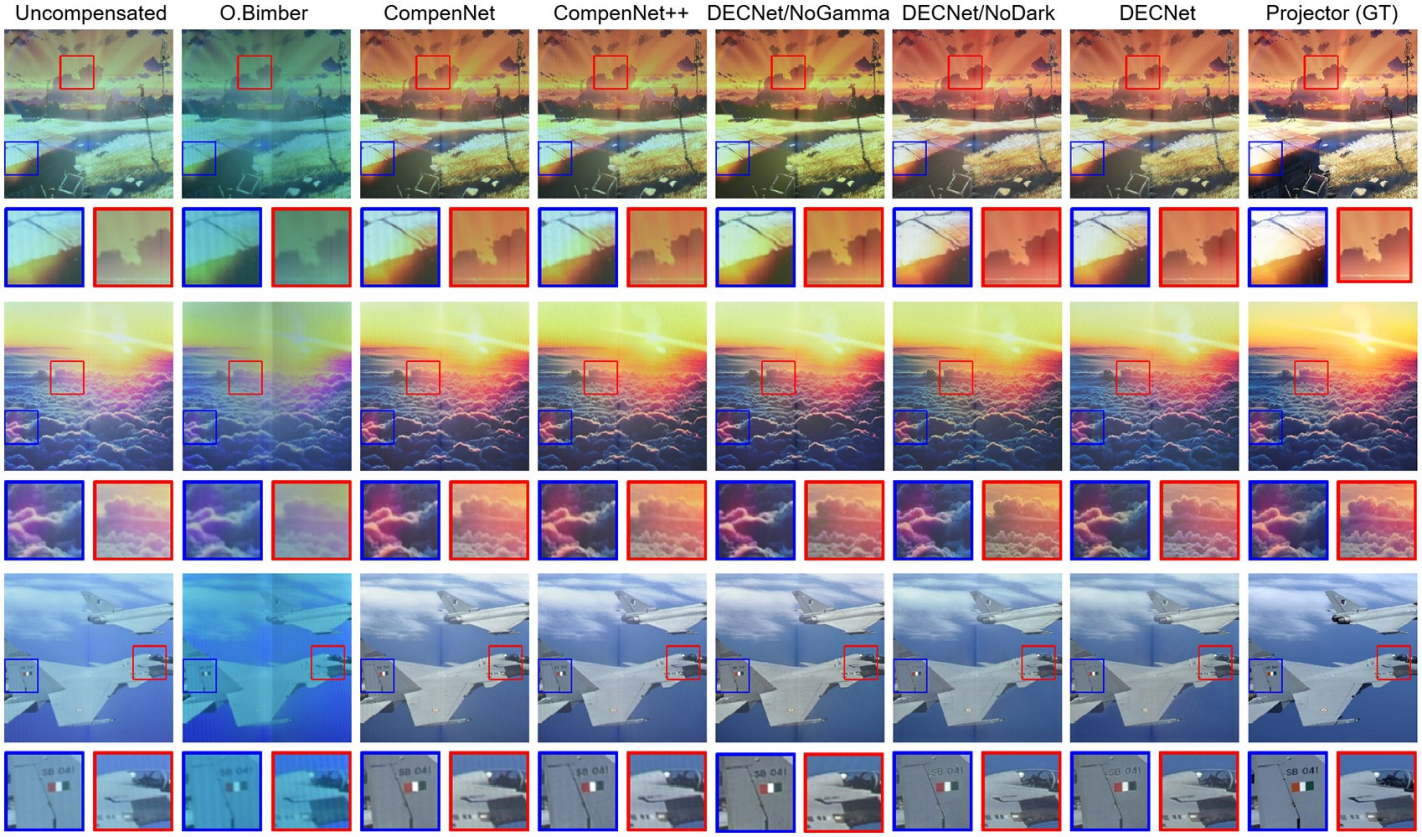
Compare O.Bimber method [19], CompenNet [10], CompenNet++ [33] and Dark-Channel Enhanced Compensation Net (DECNet) on the L-shaped screen. The first column is the uncompensated image taken by the camera. Columns 2 to 7 are the camera capture compensation results of different methods. The last column is the ground truth input image. Each image has two enlarged patches for detail comparison. Compared with DECNet, CompenNet has serious color errors. When fitting using O.Bimber’s method, they produce better results than uncompensated images, although there are still color errors and blocky effects.

Observing the map we learned in Fig 5, we can see that the design of Dark-Channel subnet is added to make the network learn the compensation intensity suitable for the foreground and background. In the comparison method, the O.Bimber’s method [19] tries to establish the mathematical model of the real projection environment and calculate the inter-reflection between different patches. O.Bimber’s method obtains all environment-related parameters, such as lens distortion, surface reflection and the detailed information of the geometric position of the projection camera. However, these parameters are difficult to obtain in real scenes due to different projection environments. Moreover, this method is harmful to the compensated image quality and projection visual effect. The experimental results are displayed in the tab. Table 1 shows the significant improvement of DECNet on O.Bimber method in traditional evaluation indicators. It can be seen that the performance of the O.Bimber method in PSNR, RMSE, and SSIM is worse than that of other methods.

**Table 1:** Quantitative analysis with different methods.

Train Model	PSNR	RMSE	SSIM
**Uncompensated**	16.8113	0.2552	0.7499
**CompenNet**	20.3879	0.1727	0.8006
**CompenNet++**	20.4886	0.1697	0.8011
**OBimber**	13.5363	0.3714	0.6711
**DECNet/NoDark**	20.5078	0.1703	0.8337
**DECNet/NoGamma**	20.8555	0.1638	0.8350
**DECNet**	**21.0798**	**0.1615**	**0.8401**

In order to prove the difficulty of the inter-reflection problem and the effectiveness of the attention mechanism after grouping normalization, we compared our method with two image-to-image translation models based on deep learning, CompenNet [10] and CompenNet++ [33], which are trained on the same data set. CompenNet and CompenNet++ compensate the projected image at the whole image level, regardless of the different illumination intensities in different areas (see Fig 5 and Table 1). Observing the results of the two models, we can see that they compensate the input image under the same-intensity. The inter-reflection in the high-intensity region can be removed well, but the low-intensity region is too dark. At the same time, the color quality of the image is greatly reduced. See Fig 5, and the standards of CompenNet and CompenNet++ compensated images are very different.

In contrast, our proposed method is superior to O.Bimbers method and CompenNet/CompenNet++ in evaluation index and visual effect. At the same time, the results show that both projection compensation and full compensation models can’t solve the inter-reflection problem well. Traditional methods, such as O.Bimbers method, have parameter and environmental reliability, and lack applicability in complex immersive projection system. The projection image quality is not as effective as DECNet. The quantitative and qualitative comparison is shown in Table 1. Our method is about 0.6 higher than CompenNet and CompenNet++ methods on PSNR, and improved SSIM performance by 5%.

### 5.2. Effective of the Gamma-corrected

In the immersive projection system, human eye observation is the most important image quality evaluation index. However, the response of human eyes to radiation is not a linear function, but a curve similar to the Gamma curve. Generally, the human eye has a greater dynamic range in shadows than in highlights, is more sensitive to low light and is less sensitive to bright light. Therefore, we fully consider this situation and Gamma-correct the image captured by the camera. Inspired by Huang. Z [37], we set the value of Gamma-corrected to 2.2, which is the value closest to the perception of human eyes. As shown in Fig 5, Gamma-corrected training datasets were compared to uncorrected images, and all conditions held constant except for Gamma-corrected. The Gamma-corrected image, can effectively weaken the inter-reflection and obtain appropriate color saturation in an immersive environment. In addition, Table 1 shows that the performance of PSNR without Gamma-corrected is slightly lower than that of our method by about 0.2. In addition, it is 1% lower than that of RMSE and SSIM. Therefore, the Gamma-corrected image obtains higher image quality, and it is proved that Gamma-corrected can effectively weaken the inter-reflection and obtain a large standard deviation in the projected image.

### 5.3. Effectiveness of Dark-Channel

In the bifurcated immersion environment, the inter-reflection light generated near the middle position is particularly strong. In view of this local light reflection imbalance, we propose the dark channel algorithm [38] to solve it, so that the compensated image is close to the original image. The depth of field image of the image is obtained by the dark channel algorithm, and the effectiveness decreases exponentially with the increase of depth of field. Therefore, we process the depth of field of the image and mix it with the original image to make the result better compensated. Make the image more realistic. As shown in Fig 5, the image optimized by the Dark-Channel is more consistent with the original image in color compensation, and higher image quality is obtained. From Table 1, it can be seen that the effect of using the DarkChannelNet on evaluation parameters is better than that of direct training, and it improved about 2.5% on PSNR, improved by about 5% on RMSE, improved SSIM performance by about 0.07. It is proved that the dark channel algorithm can effectively compensate the image and obtain better results.

### 5.4. Comparison with different loss functions

Because the most important evaluation index in the immersive projection system is human visual perception, the mainstream evaluation indexes of images are adopted, namely PSNR, SSIM, and RMSE. Therefore, we improve the loss function of Dark-Channel enhancement compensation network(DECNet) by considering the observation indexes of human eyes rather than evaluation indexes. Estimate and compare four different loss functions: L1+ssim, L1+Lssim+Lperception, L1+L2+Lssim, and L1+L2+Lssim+Lperception [40] to get the best loss.

**Fig 6:**
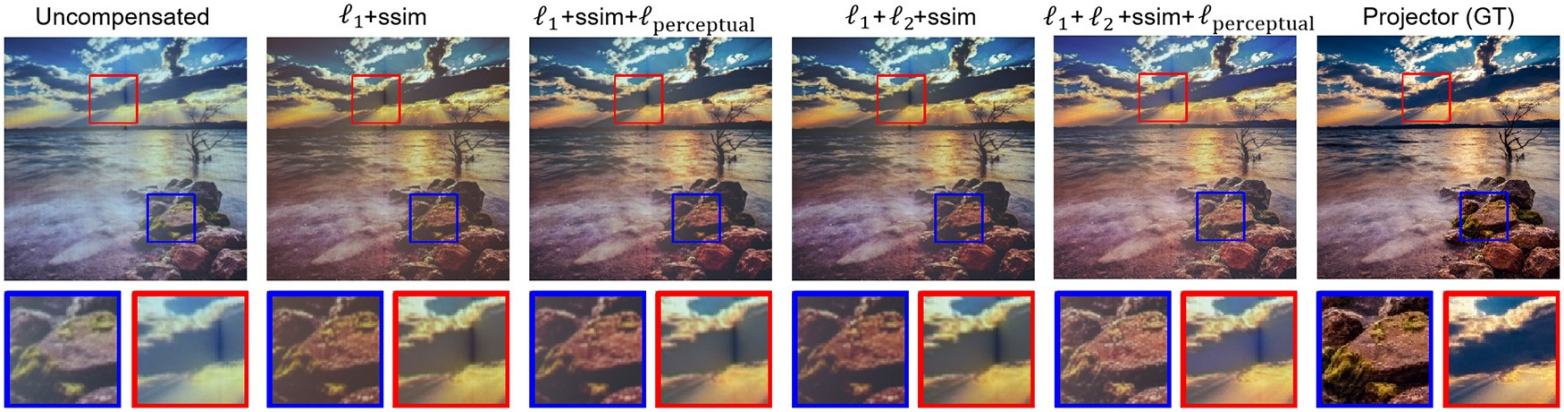
Comparison of different loss function. The 1st column is the camera-captured uncompensated projected image. The 2nd to 5th column is the camera-captured compensated different loss images. The 6th column is the ground truth original image needs to project. Each image is provided with two zoomed-inpatches for detailed comparison. Compared with 2nd, 3rd column and 4th, 5th column respectively, we can see clearly perceptual loss can improve color and details. The last column is closest to the original image.

The results are shown in Fig 6. Obviously, L1+L2+Lssim+Lperceptual has the best performance. Table 2 illustrates this advantage. In particular, the quality of color saturation or high-frequency information decreases sharply without causing perceptual loss. Therefore, loss of perception is effective in improving the performance of visual quality. We choose the best human eye sensitivity and evaluation loss function L1+L2+lssim+Lperception as the final loss function.

**Table 2:** Different loss selection of objective indicators.

Loss	PSNR	RMSE	SSIM
**l1+ssim_0.01**	20.6645	0.1707	0.8015
**l1+l2+ssim+vggLoss_0.01**	**21.0798**	**0.1615**	**0.8401**
**l1+ssim+vggLoss_0.01**	20.8776	0.1633	0.8340
**l1+l2+ssim_0.01**	20.5515	0.1698	0.8338
**Uncompensated**	16.8113	0.2552	0.7499

### 5.5. Effective of the λ

Perceptual loss can improve the edge sharpness and color saturation of projected images in an immersive environment. λ different weight values have different effects on the detail information and color saturation of the generated image, which is explained by Cycle-Dehaze [41]. We evaluated different perceptual losses 1, 0.1, 0.01, and 0.001, which controlled the effectiveness of perceptual loss.

**Fig 7:**
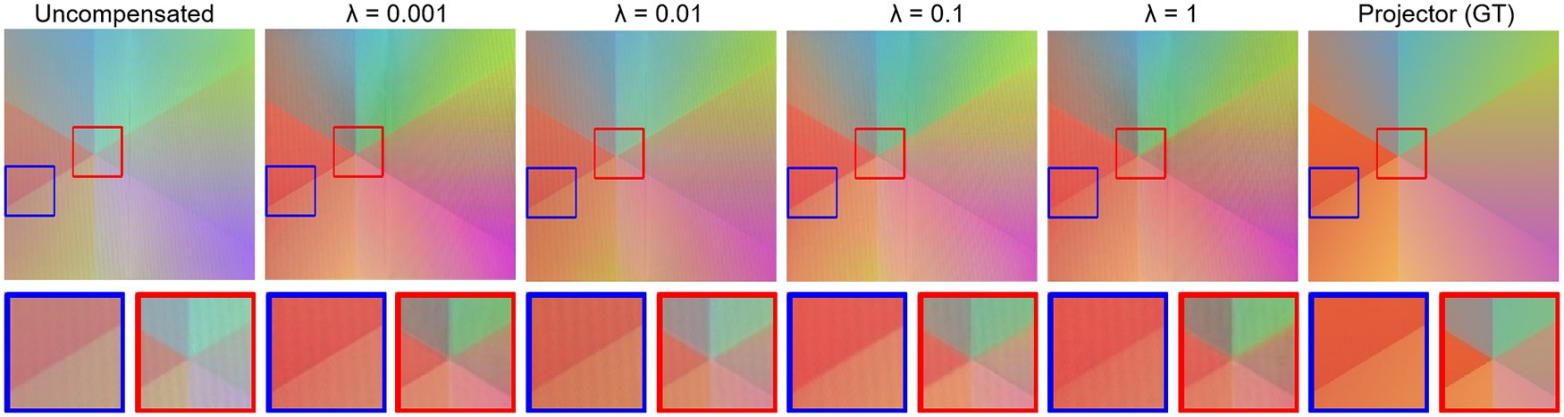
Comparison of different γ. It shows that when γ equals 1 can’t successfully compensate the inter-reflection. When γ equals 0.001, 0.01, and 0.1, it seems that they have similar results, but the picture in the blue box when γ equals 0.001 is much brighter and the flower in the red box when γ equals 0.1 is much darker. In conclusion, when γ equals 0.01,the Dark Channel Enhance-CompenNet can produce the best result.

When it is 0.01, DECNet is the best experience in people’s eyes. As shown in Fig 7 and Table 3, when in 1 and 0.1, the average illumination of the area image is too low, which means that it is over-compensated, which may reduce the quality of the projected image. At 0.01 and 0.001, DECNet seems to get the same average illumination and standard variance. But when the value is 0.01, the detailed visual effect is the best.

**Table 3:** Different λ weight parameter selection of objective indicators.

λ	PSNR	RMSE	SSIM
**λ = 0.001**	20.8004	0.1647	0.8367
**λ = 0.01**	**21.0798**	**0.1615**	**0.8401**
**λ = 0.1**	20.5005	0.1701	0.8359
**λ = 1**	21.0082	0.1614	0.8301
**Uncompensated**	16.8113	0.2552	0.7499

## 6. Conclusion

In this paper, we present an end-to-end image compensation method in immersive projection system. It’s the first time to employ the neural network-based method in compensating the inter-reflection for the immersive projection system, which is of great importance in the multi-projection VR system. In practical application, DECNet can capture the complex inter-reflection in the immersive projection system without calculating the complex light transport matrix. Moreover, in order to acquire the high visual quality of projection images, we improved the loss function by combining the pixel-wise loss function with perceptual loss. Meanwhile, we propose Gamma-corrected for the image captured by the camera instead of the directly captured image. Due to the serious problem of inter-reflection, we adopt a learnable dark channel processing method to eliminate the impact of light pollution as much as possible.

The experimental data of this paper are collected through the real projection scene, and the test data are tested in the same experimental scene to ensure the algorithm’s effectiveness. Compared with the existing algorithms, the proposed algorithms PSNR, RMSE, and SSIM have obtained the highest scores. At the same time, in terms of human visual perception, the algorithm proposed in this paper does not have serious color difference, which is in line with human visual perception. The experimental results show that our method is superior to the traditional method and the photometric compensation algorithm based on CNN in index and visual effect.

However, the algorithm in this paper has some limitations. Firstly, the DataSet compensated by the camera needs to be aligned again. Therefore, for some virtual reality devices that cannot collect the whole projected scene image with the camera, this method needs to be further improved. Secondly, the image generated by this method has the problem of decreasing the definition of some pictures, resulting in the low SSIM index. So there is still space for research to improve the definition of pictures.

## Supporting information

S1 Data (ZIP)
